# Gastric point-of-care ultrasonography in patients undergoing radical gastrointestinal surgery before anesthetic induction: an observational cohort study

**DOI:** 10.1186/s12871-024-02473-1

**Published:** 2024-03-04

**Authors:** Siming Huang, Shumei Cao, Xia Sun, Jun Zhang

**Affiliations:** 1 Department of Anesthesiology, Fudan University Shanghai Cancer Center, No.270, DongAn Road, Xuhui District, Shanghai, 200032 China; 2 Department of Oncology, Shanghai Medical College, Fudan University, No.270, DongAn Road, Xuhui District, Shanghai, 200032 China

**Keywords:** Pulmonary aspiration, Gastric point-of-care ultrasound, Gastric antrum, Gastric content, Gastrointestinal cancer

## Abstract

**Background:**

Pulmonary aspiration of gastric contents is a serious perioperative complication. Patients with gastric cancer may experience delayed gastric emptying. However, the role of qualitative and quantitative gastric ultrasound assessments in this patient population before anesthesia induction has not yet been determined.

**Methods:**

Adult patients with gastrointestinal cancer were recruited and examined using gastric point-of-care ultrasound (POCUS) before anesthetic induction from March 2023 to August 2023 in a tertiary cancer center. Three hundred patients with gastric cancer were conducted with POCUS prior to induction, and three hundred patients with colorectal cancer were included as controls. The cross-sectional area (CSA) of the gastric antrum and gastric volumes (GV) were measured and calculated. We determined the nature of the gastric contents and classified the antrum using a 3-point grading system. A ratio of GV to body weight > 1.5mL/Kg was defined as a high risk of aspiration.

**Results:**

In patients with gastric cancer, 70 patients were classified as grade 2 (23%, including 6 patients with solid gastric contents) and 63 patients (21%) were identified as having a high risk of aspiration. Whereas in patients with colorectal cancer, only 11 patients were classified as grade 2 (3.7%), and 27 patients (9.7%) were identified as having a high risk of aspiration. A larger tumor size (OR:1.169, 95% CI 1.045–1.307, *P* = 0.006), tumor located in antrum (OR:2.304, 95% CI 1.169–4.539,*P* = 0.016), gastrointestinal obstruction (OR:21.633, 95% CI 4.199–111.443, *P* < 0.0001) and more lymph node metastasis (OR:2.261, 95% CI 1.062–4.812, *P* = 0.034) were found to be positively while tumor site at cardia (OR:0.096, 95% CI 0.019–0.464, *P* = 0.004) was negatively associated with high aspiration risk in patients with gastric cancer.

**Conclusion:**

The Gastric POCUS prior to induction provides an assessment of the status of gastric emptying and can identify the patients at high risk of aspiration, especially those with gastric cancer.

**Trial registration:**

Chinese Clinical Trial Registry (www.chictr.org.cn) identifier: ChiCTR2300069242; registered 10 March 2023.

**Supplementary Information:**

The online version contains supplementary material available at 10.1186/s12871-024-02473-1.

## Introduction

Gastroesophageal regurgitation and pulmonary aspiration of gastric contents constitute formidable challenges, especially during anesthetic induction. Such an occurrence may give rise to acute respiratory obstruction and aspiration pneumonia, both of which carry a high mortality rate [1]. Notably, the majority of fasting patients who experience pulmonary aspiration have delayed gastric emptying, such as pre-existing gastrointestinal obstruction or other acute abdominal conditions [2].

Gastric cancer is the second most prevalent malignancy worldwide [3]. Dyspepsia is a common symptom observed in gastric cancer patients [4], and gastric outlet obstruction is frequently encountered in this population due to compression or invasion of the malignant tumor [5]. Despite routine fasting is adopted in clinical practice, current guidelines [6, 7] do not guarantee that these patients achieve an empty stomach state.

Gastric ultrasound, as a point-of-care tool, offers bedside qualitative and quantitative evaluation of gastric contents [8–10]. The use of gastric point-of-care ultrasound (POCUS) for the assessment of gastric volume and emptiness based on an ultrasound technique is increasing among anesthesiologists [11]. The feasibility and reliability of ultrasound assessment of gastric contents has been successfully assessed in volunteers [12], the patients admitted for elective [13] or emergency surgery [14], severely obese individuals [15], children [16], pregnant patients [17] and ICU patients [18]. It provides a quantitative assessment in ml/kg body weight for the gastric contents in addition to the qualitative assessment proposed by the adult Perlas classification [19], and allows accurately classifying the stomach as “empty” or “full”. The gastric antrum, the most suitable region of the stomach for ultrasound scanning, is highly sensitive to ultrasound imaging, and accurately reflects stomach contents [20, 21]. POCUS can also lead to changes to anesthetic management and improve individualized care, as a more flexible approach would have been possible in 15% of patients and a more conservative management necessary in 4% after pre-operative gastric ultrasound [22]. Therefore, it can be performed prior to anesthetic induction to evaluate gastric emptying and to reduce the risk of pulmonary aspiration [23].

This study aimed to examine the role of qualitative and quantitative sonographic patterns of the gastric antrum in gastric POCUS before anesthetic induction and explore the potential risk factors influencing gastric emptying in patients with gastric cancer.

## Methods

### Patients enrollment and ethics

This prospective observational cohort study compared the effectiveness of gastric ultrasound before anesthesia in patients with gastrointestinal cancer. Ethical approval for this study (Ethical number: 2210262–6) was provided by the Ethical Committee of Fudan University Shanghai Cancer Center (FUSCC), Shanghai, China (Chairperson Prof Jiong Wu) on 24 October 2022, and registered in the Chinese Clinical Trial Registry (registry number: CHICTR-2300069242; Registry URL: https://www.chictr.org.cn/showproj.html?proj=191070; principal investigator: Jun Zhang). Written informed consent was obtained from all enrolled subjects. We followed relevant guidelines and regulations, and the reporting of our study conformed to the STROBE statement [24].

**Fig. 1:**
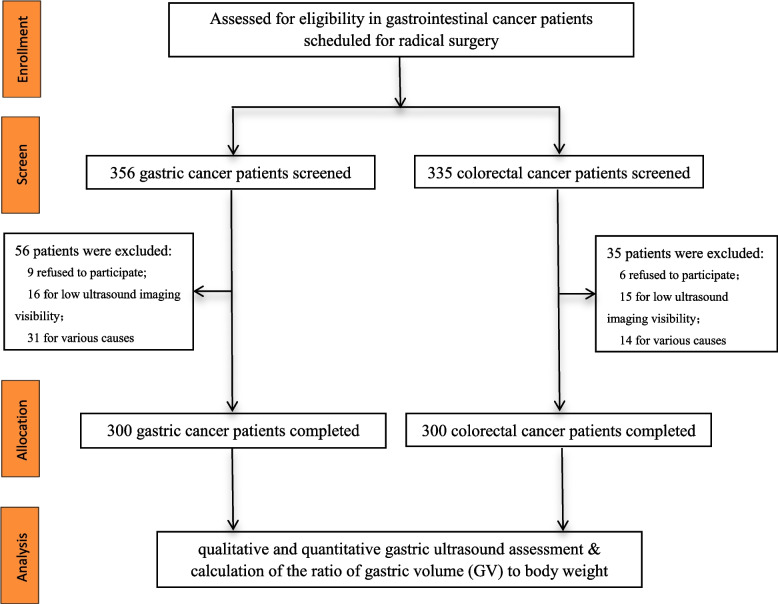
The flowchart of the study

We enrolled adult patients aged 18–80 years who were clinically diagnosed with gastric cancer or colorectal cancer by digestive endoscopy and CT examination, and scheduled for radical surgery under general anesthesia between March 2023 and August 2023 in FUSCC. The inclusion criteria were as follows (ASA) physical status I-III, preoperative fasting for at least 8 h, and the ability to understand the rationale of the study assessments. The exclusion criteria were: (1) a previous history of gastrointestinal surgery, abnormal anatomy of the upper gastrointestinal tract or indwelling gastric tube, (2) comorbid autoimmune diseases, severe neurologic conditions, hepatic disease (Child–Pugh classification C), renal failure (serum creatinine greater than 442 μmol/L), (3) medications taken that influence gastric mobility, or (4) pregnant women. The flowchart of the study is presented in Fig. 1.

### Study protocol

#### Gastric ultrasound scan

Upon entering the operating room, ultrasonography was performed by an experienced anesthesiologist (Huang S) using a color Doppler ultrasound system (Venue™, GE Healthcare, USA) for gastric assessment. Each ultrasound examination was conducted with the patient's head elevated at 45° in the supine position, followed by the right lateral decubitus (RLD) position. Imaging of the antrum was performed in the parasagittal plane in the epigastric area using the left lobe of the liver, inferior vena cava, and superior mesenteric vein as internal landmarks, which typically appeared slightly to the right of the abdominal midline. A detailed description of the technique and sonographic characteristics of the gastric antrum content has been previously reported [12]. Qualitative assessment of the gastric antrum aimed to determine the nature of the gastric contents (empty, fluid, or solid content) and classify patients according to the Perlas three-point qualitative grading system [19]. Grade 0 is defined as an empty antrum in both the supine and RLD positions, while Grade 1 is characterized by fluid content observed only in the RLD position, indicating a small fluid volume. Grade 2 represents the fluid content observed at both positions, which suggests a high volume state (over 100ml of gastric fluid in 75% of cases) [25]. In our study, the solid content was rated grade 2.

**Fig. 2:**
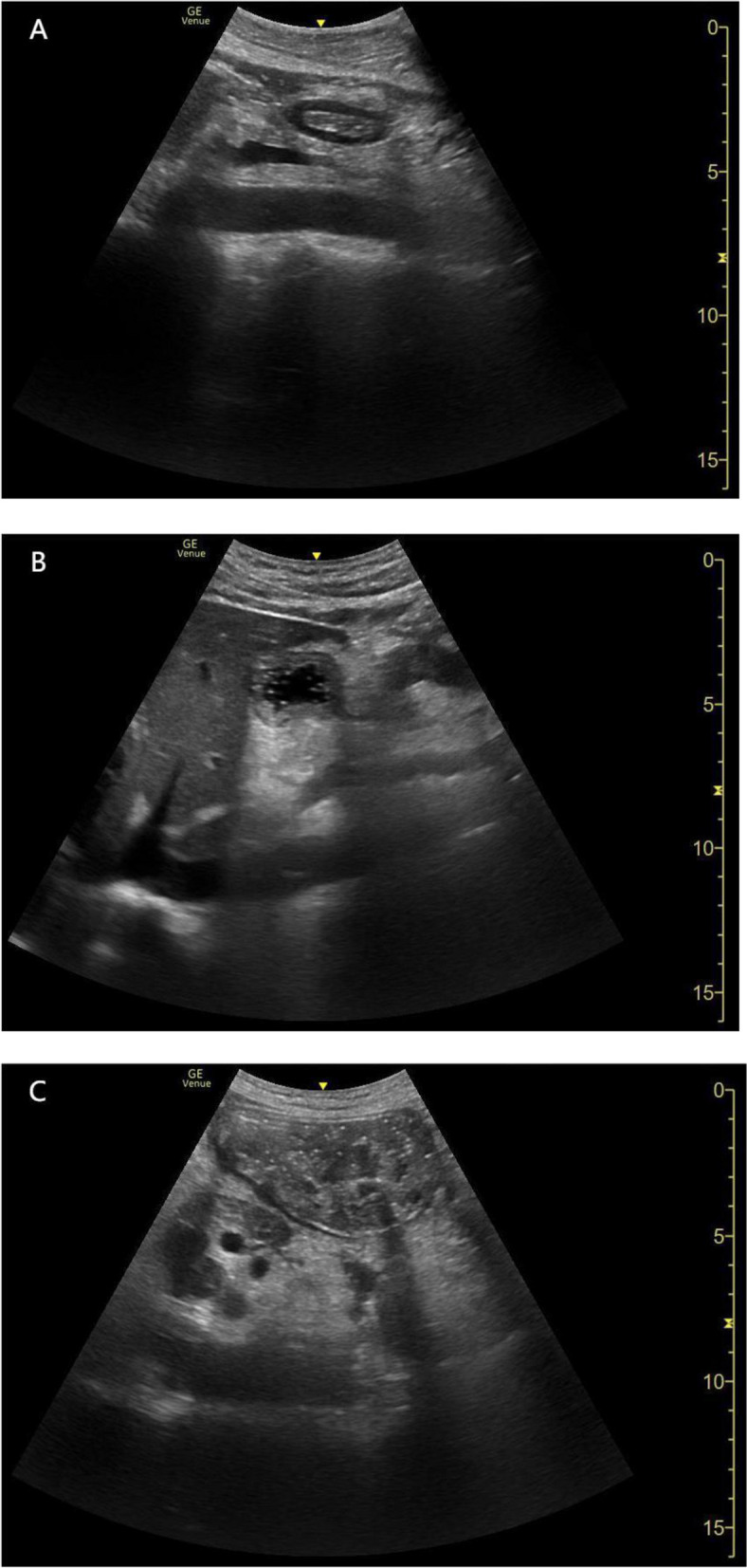
Sonographic images of the epigastric area with the gastric cancer patients in the right-lateral decubitus. Antrum: (**a**) empty; (**b**) fluid; (**c**) solid content

The gastric volume was quantitatively assessed by measuring the gastric antrum. Once these vessels were identified as internal landmarks, the transducer was rotated slightly clockwise or counterclockwise to obtain a true cross-sectional view of the antrum (smallest possible cross-sectional view; Fig. 2). The anteroposterior (D1) and craniocaudal (D2) diameters between the serosal surfaces of the gastric antrum were measured. Two measurements were used to calculate the cross-sectional area (CSA) of the gastric antrum in the supine and RLD positions using the formula described by Bolonde [26]:

$$\mathbf{C}\mathbf{S}\mathbf{A}\,({\mathbf{c}\mathbf{m}}^{2})=({\varvec{\uppi}}\times \mathbf{D}1\times \mathbf{D}2)/4$$

We also used the free-trac in caliper of the ultrasound unit to measure the CSA of the gastric antrum. This method of area measurement was easy and highly reproducible [27]. We then used the formula adopted by Perlas to predict gastric volume (GV) [25]:

$$\mathbf G\mathbf V\left(\mathbf m\mathbf l\right)=27.0+14.6\boldsymbol\,\ast{\boldsymbol\,\mathbf{CSA}}_{\mathbf{RLD}}\,\left({\mathbf c\mathbf m}^2\right)-1.28\boldsymbol\,\ast\,\mathbf a\mathbf g\mathbf e\,(\mathbf y\mathbf r\mathbf s)$$

Where CSA_RLD_ represents CSA of gastric antrum in the RLD position.

This formula has been applied to non-pregnant adults with a BMI of less than 40 kg/m^2^, which can predict gastric volume from 0 to 500 ml with relative accuracy. A GV to body weight ≤ 1.5 ml/kg (approximately 100 ml on average) was associated with a lower risk of reflux aspiration [9, 28, 29]. In this study, high aspiration risk was defined as GV/weight > 1.5 ml/kg of liquid substance or visible solid material in the gastric antrum, also called a “full stomach.”

All patients at risk of gastric regurgitation were managed in accordance with the anesthetic management of perioperative “full stomach”, namely, a rapid sequence induction (RSI) strategy was applied during anesthetic induction. In contrast, those patients without “full stomach” were managed with routing anesthetic induction.

#### Data collections

We reviewed medical records documented and recorded demographic and clinical characteristics, including preoperative surgical diagnosis; preoperative history of chemotherapy, radiation therapy, or immune therapy; medical comorbidities; previous surgery; personal history (smoking and drinking); laboratory examination (albumin and glycated albumin); and pathologic diagnosis and tumor-node-metastasis (TNM) stage. We also asked patients about their digestive symptoms (ie. Nausea, vomiting and obstruction). We also confirmed the fasting time with the patients, family members and the nurse. Preoperative gastrointestinal obstruction was diagnosed by gastroscopy or CT examination.

The primary outcome of this study was the incidence of antrum grade 2 in the enrolled fasting patients with gastrointestinal cancer at term. Secondary outcomes included (1) the incidence of antrum grade 0 and 1 in both groups; (2) the antral CSA measurements in both examination positions; (3) an estimate of GV based on the antral CSA in the RLD and the incidence of the high aspiration risk; (4) risk factors for predicting a “full stomach.”

### Bias

The risk of selection bias was controlled by consecutive enrolment of patients. To avoid detection bias, ultrasonography was performed by a specialized anesthesiologist (Huang S). And fasting times were not only dictated by the patients but also checked with the nurses to minimize the risk of information bias.

### Statistical analysis

The incidence of grade 2 antrum in fasted surgical patients was 3.5% [20], and in our pretest with gastric cancer patients was 10%. With the alpha set at 0.05, and power at 85%, a sample size of 528 participants (264 per group) was required, at least estimated using PASS 2021 sample size software (NCSS, LLC). We enrolled 600 patients (300 per group), considering a dropout rate of 12%.

For continuous variables, data are expressed as mean ± standard deviation (SD) or median (interquartile range). If the data followed a normal distribution, intergroup comparisons were conducted using an independent-sample *t* test. If the data did not follow a normal distribution, intergroup comparisons were performed using the Mann–Whitney U test. For categorical variables, data were described using frequency (composition ratio) and analyzed using Pearson’s chi-square test. Multivariate analysis was performed by logistic regression using a backward (likelihood ratio) method, considering variables that achieved statistical significance in univariate analysis to identify the factors associated with high aspiration risk. We adjusted for age, sex, BMI, ASA physical status and diabetes, which may affect gastric emptying. Multicollinearity was assessed before the final model was established. The receiver operating characteristic (ROC) curve was plotted to test the efficacy of the regression models, and the area under the ROC curve (AUC) was calculated. For all hypotheses, two-tailed P values < 0.05 were considered statistically significant. All statistical analyses were performed using SPSS software (26.0 version, IBM SPSS, USA).

## Results

A total of 356 patients who underwent radical gastric cancer resection under general anesthesia were recruited and evaluated. Among them, 16 patients were excluded for low ultrasound imaging visibility due to abdominal fat thickening, which made it difficult to identify the gastric antrum and accurately determine the gastric contents; 9 patients refused to participate; and 31 patients were excluded for various reasons, including 10 patients with a detained gastric tube, 6 patients with residual gastric cancer who underwent reoperation, and 15 patients were canceled for surgery as scheduled. In addition, 335 patients with colorectal cancer were included in the control group, in which 15 patients were excluded for low ultrasound imaging visibility, 6 patients refused to participate, and 14 patients were canceled for surgery as scheduled. Thus, 600 patients (*n* = 300 in each group) were included in the final analysis.

The demographic and clinical characteristics of patients are shown in Table 1. The demographics of the two groups were comparable. ASA physical status and the incidence of diabetes mellitus (DM) were also similar in both groups. However, the incidence of gastrointestinal obstruction in patients with gastric cancer (13/300) was significantly higher than that in patients with colorectal cancer (0/300; *P* < 0.0001). Furthermore, gastric cancer patients (for solids was 34 (24, 38), and for clear liquids was 11 (10, 12)) had longer fasting durations than colorectal cancer patients (for solids was 24 (23, 36), *P* ≤ 0.0001, and for clear liquids was 11 (10, 12), *P* = 0.001, respectively).

**Table 1:** Demographics and clinical characteristics (*n* = 300/group)

	**Gastric cancer**	**Colon cancer**	***P* value**
Age, yrs	60.3 ± 11.6	62.0 ± 11.2	0.057
Male sex, n (%)	188 (62.7)	170 (56.7)	0.134
Weight, kg	64.0 ± 11.2	63.4 ± 11.0	0.498
Height, cm	165.5 ± 7.4	165.4 ± 8.0	0.865
BMI, kg/m^2^	23.3 ± 3.2	23.1 ± 3.2	0.529
ASA physical status, n (%)			0.087
I	172 (57.3)	148 (49.3)	
II	125 (41.7)	145 (48.3)	
III	3 (1.0)	7 (2.3)	
Diabetes, n (%)	44 (14.7)	49 (16.3)	0.573
Gastrointestinal obstruction, n (%)^a^	13 (4.3)	0 (0)	< 0.0001
Fasting for solids, h	34 (24, 38)	24 (23, 36)	< 0.0001
Fasting for clear liquids, h	11 (10, 12)	11 (10, 12)	0.001

The data are expressed as mean ± SD, n (%) or median (interquartile range)

*BMI* body mass index

^a^Gastrointestinal obstruction means that the patients have a preoperative gastrointestinal obstruction known by gastroscopy or CT examination

According to the gastric ultrasound measurements, the gastric antrum classifications in both groups are shown in Table 2. Gastric antrum CSA and predicted volume were similar between the patients with gastric cancer and colon cancer. Among the patients with gastric cancer, 92 patients (31%) were classified as grade 0, 138 patients (46%) as grade 1, and 70 patients (23%) as grade 2. Specially, 6 patients was found solid contents in their stomachs, among them 3 patients were diagnosed as gastrointestinal obstruction by preoperative imaging methods. Whereas according to the ratio of GV to body weight (> 1.5 ml/kg), a total of 63 patients (21%) were identified as being at high risk of aspiration. Interestingly, 2 of 13 patients diagnosed as gastrointestinal obstruction by gastroscopy or CT examination was identified by POCUS as low risk of aspiration (GV/body weight < 1.5 ml/kg). Among the patients with colorectal cancer, 130 patients (43.3%) were classified as grade 0, 159 patients (53.0%) as grade 1, and 11 patients (3.7%, 1 patient with solid gastric contents) as grade 2. In addition, 27 patients (9.7%) were identified to have a high risk of aspiration (> 1.5 ml/kg).

**Table 2:** Ultrasound-measured antral area and gastric volume by the gastric antral grades

	**Gastric cancer**	**Colon cancer**	***P* value**
**Grade 0**	***n* = 92**	***n* = 130**	
CSA in supine, cm^2^	2.7 (2.0, 3.4)	2.9 (2.4, 3.7)	0.067
CSA in RLD, cm^2^	4.1 (3.3, 5.3)	4.2 (3.6, 5.1)	0.980
Predicted volume, mL^a^	16.4 (0, 42.1)	11.9 (0, 29.1)	0.088
Predicted volume, mL/kg	0.3 (0, 0.7)	0.2 (0, 0.4)	0.084
**Grade 1**	***n* = 138**	***n* = 159**	
CSA in supine, cm^2^	3.4 (2.6, 4.8)	3.7 (3.0, 4.6)	0.312
CSA in RLD, cm^2^	7.5 (5.7, 9.2)	7.1 (6.1, 8.6)	0.594
Predicted volume, mL^a^	55.6 (32.6, 83.1)	48.3 (31.4, 75.8)	0.216
Predicted volume, mL/kg	0.9 (0.5, 1.3)	0.8 (0.5, 1.1)	0.197
**Grade 2**	***n* = 70**	***n* = 11**	
CSA in supine, cm^2^	6.3 (4.6, 8.9)	5.3 (4.3, 6.5)	0.247
CSA in RLD, cm^2^	10.5 (7.8, 14.5)	9.4 (8.1, 12.2)	0.370
Predicted volume, mL^a^	106.5 (59.5, 166.6)	85.7 (76.9, 118.8)	0.385
Predicted volume, mL/kg	1.7 (0.9, 2.6)	1.3 (1.1, 1.9)	0.356

The data are expressed as median (interquartile range)

*CSA* antral cross-sectional area, *RLD* right lateral decubitus position

^a^Predicted volumes (mL) based on a mathematical model previously validated

According to preoperative defined protocol, 13 patients (2.2%) diagnosed as gastrointestinal obstruction should undergo anesthetic induction with RSI protocol. Actually, among them 2 patients changed from defined RSI protocol into routing induction protocol; and a total of 90 patients (15%, including 4 patients with gastric solid contents while without gastrointestinal obstruction) underwent anesthetic induction with RSI protocol, namely, additional 79 patients changed from defined routing induction protocol into RSI protocol after POCUS examination. Fortunately, no clinical aspiration occurred in all of these patients.

Since more patients with gastric cancer were at a high risk of pulmonary aspiration than those patients with colorectal cancer, subsequently, the risk factors for “full stomach” in gastric cancer patients were further analyzed. The results of univariate analysis for high aspiration risk in patients with gastric cancer are shown in Supplementary Table 1. The results demonstrated that tumor size, plasma albumin level, preoperative gastrointestinal obstruction and vomiting, and TNM stage were associated with a larger GV to body weight (> 1.5 ml/Kg), suggesting a high aspiration risk.

**Fig. 3:**
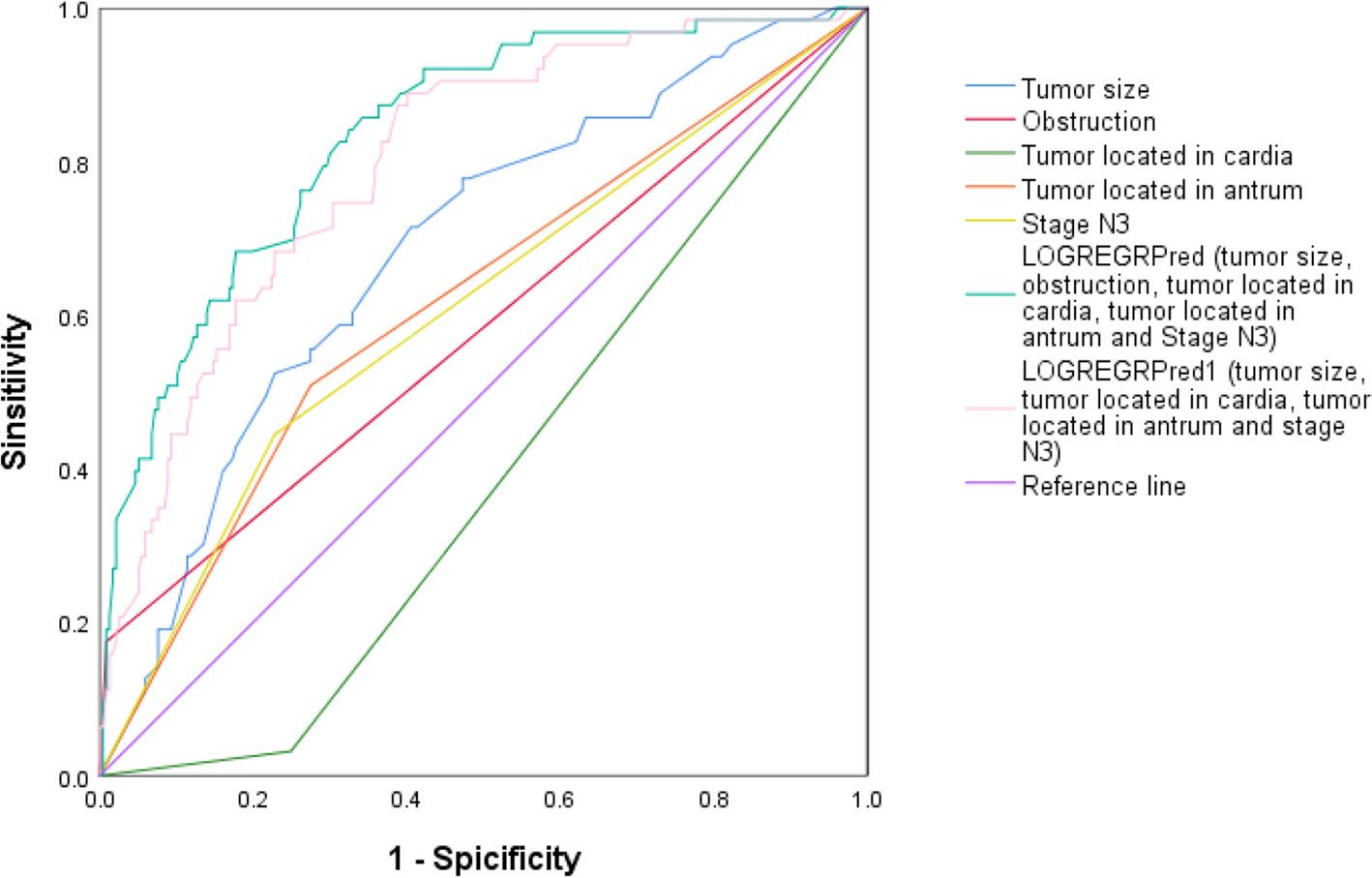
The ROC curve for predicting high aspiration risk in patients with gastric cancer derived from tumor size, gastrointestinal obstruction, tumor located in cardia, tumor located in antrum, N3 stage, logistic regression predicted probability using the above five variables (tumor size, obstruction, tumor located in cardia, tumor located in antrum, N3 stage) and logistic regression predicted probability using the above four variables (tumor size, tumor located in cardia, tumor located in antrum and N3 stage). Area under ROC curve (AUC) were 0.687 (95% CI 0.615–0.759), 0.583 (95% CI 0.498–0.668), 0.391 (95% CI 0.321–0.462), 0.617 (95% CI 0.536–0.697), 0.617 (95% CI 0.536–0.697), 0.608 (95% CI 0.527–0.690), 0.835 (95% CI 0.780–0.890) and 0.798 (95% CI 0.739–0.857), respectively. CI = confidence interval

Multivariate logistic regression analysis was performed to identify the risk factors for aspiration of gastric contents in patients with gastric cancer (Table 3). There was no significant multicollinearity between independent variables. The factors distinguishing the patients with a high risk of aspiration from those with a low risk after adjusting for age, sex, BMI, ASA physical status, and diabetes (may affect gastric emptying) were tumor size, tumor located at the cardia, tumor located at the antrum, gastrointestinal obstruction, and N3 stage, which resulted in an AUC of 0.835 (95% CI 0.780–0.890) for predicting a high aspiration risk (Fig. 3). The ROC curve was also plotted to test the efficacy of tumor size, tumor located at the cardia or antrum, and N3 stage, and the AUC was 0.798 (95% CI 0.739–0.857). In contrast, tumors located in the cardia were recognized as protective factors against a high aspiration risk.

**Table 3:** Multivariate logistic regression analysis of high aspiration risk in patients with gastric cancer

Variables	β value	OR (95% CI)	*P* value
Tumor size	0.156	1.169 (1.045–1.307)	0.006
Gastrointestinal obstruction	3.074	21.633 (4.199–111.443)	< 0.0001
Tumor located at cardia	-2.361	0.096 (0.019–0.464)	0.004
Tumor located at antrum	0.835	2.304 (1.169–4.539)	0.016
Stage N3	0.816	2.261 (1.062–4.812)	0.034

*OR* odds ratio, *CI* confidence interval

## Discussion

Gastric POCUS is a safe and noninvasive bedside tool recommended in children undergoing elective surgery when fasting instructions have not been applied and in children undergoing emergency surgery by the European Society of Anesthesiology and Intensive Care [30]. However, ultrasound assessment of gastric contents and volume may be also useful for our clinical decision-making in those adult surgical patients who potentially have impaired gastric emptying if the equipment is available [31]. By using gastric POCUS, our study assessed preoperative gastric conditions qualitatively and quantitatively in fasting patients with gastrointestinal cancers. As demonstrated by the findings of the present study, a “full stomach” is more common in fasting patients with gastric cancer than in those with colorectal cancer, which leads to a change in planned anesthetic induction protocol in those ones who were not previously identified as high risk of aspiration. Besides screening by gastric POCUS scans, four risk factors, including larger tumor size, tumor at the antrum, more lymph node metastasis (N3 stage), and preoperative gastrointestinal obstruction, was identified as a classifier of “full stomach” in gastric cancer patients. Thus, our study provides evidence that gastric POCUS can inform us the risk of aspiration in patients with gastrointestinal cancer, particularly in those with gastric cancer.

In this study, the incidence of grade 2 gastric antrum in patients with gastric cancer (23%) was far higher than that reported in adult surgical patients (3.5%)^19^, obese patients (2%) [32], and pregnant women (1%) [17]. Interestingly, even after excluding patients with known gastrointestinal obstruction, the incidence of grade 2 was still as high as 19%. However, the incidence of grade 2 gastric antrum in colorectal cancer patients (3.7%) was similar to that reported in previous studies [17, 19, 32, 33]. These data suggests the incidence of gastric cancer patients who had a large volume was much higher than that in fasting non-gastric cancer patients. Moreover, we observed that 6 patients with gastric cancer had solid gastric contents prior to induction, including 3 patients without preoperative known digestive obstruction, much more than that reported in previous studies [20, 26], which reminds us that gastric cancer patients are at high risk of aspiration. Local lesions in patients with gastric cancer may include luminal stenosis, stiffened gastric wall, reduced gastric motility, and even gastric outlet obstruction in more severe cases [5],. Gastric cancer is also associated with delayed gastric emptying [34]. Malignant gastroparesis is commonly observed in patients with gastric cancer and leads to delayed gastric emptying in the absence of mechanical obstruction [35]. Malignant infiltration of the autonomic nervous system and destruction of the enteric nervous system-mediated dysmotility may be associated with malignant gastroparesis. This explains why gastric cancer patients have a higher incidence of a larger gastric volume.

Our quantitative and qualitative POCUS evaluation of the antrum showed that the actual values of antral CSA measured in the supine and RLD positions were very similar to those obtained in previous studies in terms of the same Perlas grades of gastric antrum [20, 26], suggesting the gastric POCUS can also identify those high-risk of aspiration in our study populations. Preoperative gastric conditions can be acquired by other prior imaging modalities, such as CT scan, however, POCUS can be easily and dynamically applied at the bedside, even the gastric and fasting conditions have changed immediately before anesthesia. Consequently, after POCUS scan, the risk of aspiration has been re-assessed. Especially, those patients without preoperative gastrointestinal obstruction by imaging information were found to be “full stomach”, and the pre-determined anesthetic induction protocol changed to ensure safety, further suggesting a precise role of gastric POCUS examination in gastrointestinal cancer patients. The incidence of perioperative pulmonary aspiration ranges from 0.015% up to 0.17% over the last two decades [36–38]. There was no occurrence of aspiration in present study, which may benefit from POCUS. Giving the serious consequences of pulmonary aspiration, there is no study so far that takes the risk of aspiration without intervention (ie. Sellick maneuver) when a “full stomach”is found before anesthetic induction. Therefore a well-designed clinical trial with a large-size population is needed in the future to confirm or refute the benefit of ultrasound-guided anaesthetic strategy on patient outcome in terms of reducing the incidence of pulmonary aspiration.

As we all know, gastrointestinal obstruction has long been recognized as is one of a high aspiration risk. In this study, by using POCUS, we found that gastrointestinal obstruction is associated with a high aspiration risk. Obviously, these patients should be treated as the “full stomach” regardless of fasting duration, emphasizing that fasting time could not substitute for ultrasound measurements [39]. Furthermore, A previous study showed that patients with gastric outlet obstruction had a larger tumor size than those without gastric outlet obstruction [40]. Patients with gastric outlet obstruction had more lymph node metastases than those without gastric outlet obstruction [41]. Although gastric outlet obstruction is not equivalent to a high aspiration risk, it also shows that altered structure and function of the stomach is expected when a larger tumor size and more lymph node metastasis are observed, which suggests delayed gastric emptying. Interestingly, tumor at cardia was found as a protective factor for delayed gastric emptying when compared to other locations of gastric cancer lesions, which may be because the cardia is located above the stomach and has less of an effect on gastric peristalsis and emptying. Nevertheless, a tumor located above the cardia also prevents food entering the stomach, there’s a possibility of food remaining in the esophagus, although an “empty stomach.”

The gastric cancer patients fasted longer than those colorectal cancer patients, as directed by their scheduled surgery. However, preoperative fasting does not guarantee an empty stomach, and there is no observed association between aspiration and compliance with the common fasting guidelines [42]. This study also found that in the univariate analysis, the occurrence of a “full stomach” was significantly higher in patients who had preoperative vomiting than in those without vomiting symptoms, since vomiting can be a symptom of gastrointestinal obstruction [43]. Nevertheless, after multivariate logistic regression analysis, preoperative vomiting was no longer a contributing factor for high aspiration risk. Although it is essential to pay attention to whether patients have preoperative vomiting when assessing patients with gastric cancer before anesthesia, the reasons for vomiting may vary, such as preoperative chemotherapy.

Our study had several limitations. First, the formulas used to calculate gastric CSA and GV in this study and the upper limit of the safe cut-off for risk of aspiration (1.5ml/kg) were derived from previous studies on healthy adults, and it is not known whether they are also reliable for the patients with distorted gastric anatomy or compliance (both can happen in gastric cancer). Second, since benign gastroparesis is commonly observed in DM patients [44], which is also considered a risk factor for delayed gastric emptying [45]; however, we cannot identify this due to the limited numbers of DM patients in our study. Third, the fasting duration in some patients was longer than that recommended by guidelines [6, 7], although fasting time was not associated with antral CSA in Dupont’s study [43] nor associated with a high aspiration risk in our study. And finally, due to ethic issue, preventive intervention (ie. RSI) was adopted during anesthetic induction for those “full stomach”patients, we did not know how many at “high-risk”patients did in fact go on to aspirate. Therefore we could not determine the risk–benefit of gastric POCUS prior to induction.

## Conclusions

In summary, “full stomach”is common in fasting patients with gastrointestinal cancer. This potentially places these patient populations, especially gastric cancer patients, at an increased risk of aspiration of the gastric contents. POCUS can be used to qualitatively and quantitatively assess preoperative gastric content and volume, and provides objective and personalized assessment of aspiration risk although its benefit in reducing pulmonary aspiration has not been clinically validated. Importantly, we can identify those patients who are at a high risk of aspiration. Further, several risk factors may be useful, which need to verify with a larger sample size, for screening out those patients who are at high aspiration risk before anesthetic induction even if POCUS is not available.

### Supplementary Information

**Supplementary Material 1.**

## Data Availability

The datasets used and/or analysed during the current study are available from the corresponding author on reasonable request.

## Abbreviations

POCUS: Point-of-care ultrasound
ASA: American Society of Anesthesiologists
TNM: Tumor-node-metastasis
RLD: Right lateral decubitus
CSA: Cross-sectional area
GV: Gastric volume
BMI: Body mass index
ROC: Receiver operating characteristic
