# Supplementary material for: Gastric point-of-care ultrasonography in patients undergoing radical gastrointestinal surgery before anesthetic induction: an observational cohort study
Source: BMC Anesthesiol. 2024 Mar 4;24:90. doi: 10.1186/s12871-024-02473-1 (PMC10910806; doi:10.1186/s12871-024-02473-1)
Supplement: Supplementary file 1 — Supplementary Material 1. [file 12871_2024_2473_MOESM1_ESM.docx]

| **Supplementary Table 1. Univariate analysis for high aspiration risk in patients with gastric cancer** | | | | |
| --- | --- | --- | --- | --- |
| **Variables** | | **GV/Weight>1.5ml/kg (n=63)** | **GV/Weight<1.5ml/kg (n=237)** | **P value** |
| Age, yrs | | 60.19 ± 10.9 | 60.27 ± 11.7 | 0.959 |
| Male sex, n (%) | | 41 (65.1) | 147 (62.0) | 0.656 |
| BMI, kg/m^2^ | | 23.19 ± 2.9 | 23.3 ± 3.3 | 0.813 |
| Fasting for solids (h) | | 37 (24, 38) | 25 (24, 38) | 0.383 |
| Fasting for liquids (h) | | 11 (11, 13) | 11 (10, 12) | 0.188 |
| Diabetes, n (%) | | 14 (22.2) | 30 (12.7) | 0.060 |
| Albumin, g/l | | 40.0 ± 5.3 | 42.4 ± 3.6 | <0.0001 |
| Glycated albumin, % | | 14.1 ± 3.9 | 14.4 ± 2.6 | 0.479 |
| Tumor size, cm | | 6.0 (3.5, 8.0) | 3.0 (2.0, 5.0) | <0.0001 |
| Gastrointestinal obstruction, n (%) | | 11 (17.5) | 2 (0.8) | <0.0001 |
| Preoperative vomiting, n (%) | | 9 (14.3) | 12 (5) | 0.015 |
| Preoperative chemotherapy, n (%) | | 4 (6.3) | 25 (10.5) | 0.321 |
| Tumor site |  |  |  | <0.0001 |
|  | Cardia, n (%) | 2 (3.2) | 59 (24.9) | 0.002 |
|  | body of stomach, n (%) | 14 (22.2) | 73 (30.1) | 0.185 |
|  | antrum, n (%) | 32 (50.1) | 65 (27.4) | 0.001 |
|  | angular incisure, n (%) | 7 (11.1) | 20 (8.4) | 0.511 |
|  | multiple lesions, n (%) | 8 (12.7) | 20 (8.4) | 0.305 |
| ASA physical status | |  |  | 0.323 |
|  | I, n (%) | 31 (49.2) | 141 (59.5) |  |
|  | II, n (%) | 31 (49.2) | 94 (39.7) |  |
|  | III, n (%) | 1 (0.16) | 2 (0.8) |  |
| T stage |  |  |  | 0.002 |
|  | T1, n (%) | 13 (20.6) | 85 (35.9) | 0.024 |
|  | T2, n (%) | 2 (3.2) | 32 (14.8) | 0.036 |
|  | T3, n (%) | 12 (19.0) | 39 (16.5) | 0.627 |
|  | T4, n (%) | 36 (57.1) | 81 (34.2) | 0.001 |
| N stage |  |  |  | 0.001 |
|  | N0, n (%) | 18 (28.6) | 121 (51.1) | 0.002 |
|  | N1, n (%) | 5 (7.9) | 31 (13.1) | 0.269 |
|  | N2, n (%) | 12 (19.0) | 31 (13.1) | 0.232 |
|  | N3, n (%) | 28 (44.4) | 54 (22.8) | 0.001 |
| M stage |  |  |  | 0.019 |
|  | M0, n (%) | 52 (82.5) | 219 (92.4) | 0.082 |
|  | M1, n (%) | 11 (17.5) | 18 (7.6) | 0.022 |

The data are expressed as mean ± SD, median (interquartile range), or n (%). BMI = body mass index.

T1 tumor has invaded the mucosa or submucosa layer; T2 tumor has invaded the muscular layer; T3 tumor has invaded the subserosa; T4 tumor has invaded the serosa or adjacent organs; N0 no regional lymph node metastasis; N1 1-2 regional lymph node metastasis; N2 3-6 regional lymph node metastasis; and N3 ≥ 7 regional lymph node metastasis; M0 cancer has not spread to other parts of the body; M1 cancer has spread to other parts of the body.
